# Decoding the interplay between protic ionic liquids and drug: spectrophotometric, electrochemical, and DFT exploration of mitoxantrone

**DOI:** 10.1039/d5ra05841d

**Published:** 2026-02-19

**Authors:** Arti Sharma, Ravinder Sharma, Ritu, Pamita Awasthi, Indra Bahadur

**Affiliations:** a Department of Chemistry, National Institute of Technology Hamirpur-177005 India sharmaravinder444@gmail.com; b Department of Chemistry, Dr B R Ambedkar National Institute of Technology Jalandhar-144011 India; c Department of Chemistry, North-West University Mafikeng Campus Mmabatho 2735 South Africa bahadur.indra@nwu.ac.za

## Abstract

Thus far, ILs have attracted growing interest in pharmaceutical applications due to their systematic variation of physicochemical properties that can be tailored to overcome the relevant formulation hurdles associated with poorly soluble and unstable drugs. Beyond solvents, ILs could serve as media for stabilization or even as co-excipients to provide a versatile matrix for enhanced drug performance with decreased side effects. Although most anthracycline antibiotics, such as daunomycin and adriamycin, are cardiotoxic, others, such as mitoxantrone (MTX), are effective and less cardiotoxic. Because it has a planar tricyclic chromophore, MTX, with its protonated side chains, could potentially be involved in dynamic noncovalent interactions, particularly at the level of nucleic acids. In this work, we investigated the molecular interactions of MTX with three protic ILs, namely, ethanol ammonium acetate (EAAc), ethanol ammonium butyrate (EABu), and ethanol ammonium hexanoate (EAHx), using a combination of various techniques including spectroscopic, electrochemical and computational methods. In the UV-visible spectrum, the hypochromic effects observed with the addition of ILs indicate changes in the electronic environment around MTX. The corresponding fluorescence measurements demonstrated enhanced emission intensity with the formation of stable MTX–IL aggregates. Electrochemical studies unambiguously demonstrated an apparent effect of ionic liquid's alkyl chain length on binding affinity, with EAHx displaying the strongest interaction, as evidenced by the higher binding constants and lower Gibbs free energy changes. The experimental results were further supported by density functional theory calculations, indicating an enhancement in the thermodynamic stability and electrostatic complementarity of the MTX–EAHx complex. This combined evidence highlights protic ionic liquids as potential excipients for enhancing the solubility, stability and delivery efficiency of mitoxantrone, with potential for the formulation of safer and more efficient IL-assisted formulations against cancer.

## Abstract 2

Interaction between Protic Ionic Liquids (PIL) and mitoxantrone (MTX) using different techniques.

## Graphical abstract

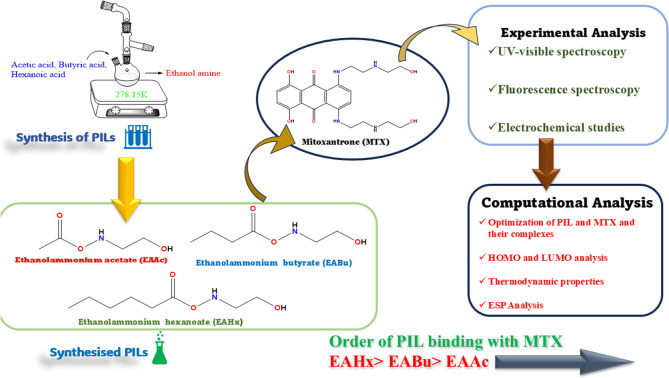

## 1. Introduction

A key challenge in the pharmaceutical industry is improving the performance of active pharmaceutical ingredients. Many APIs exhibit low water solubility, poor bioavailability, low permeation through the gastrointestinal tract lining, and poor stability in physiological conditions. Such inherent restrictions generally diminish the therapeutic responses, and accordingly, this would demand the therapeutically administered dose to be increased, which would eventually lead to an increased risk of systemic toxicity.^1–3^ As a result of this, strategies to address these issues have been the aim of the most recent works in contemporary drug formulation research, and considerable resources are being made available for developing methods that enhance the solubility, stability, and targeted capabilities of poorly soluble/hydrophobic drugs. In this context, ionic liquids (ILs) have emerged as promising functional materials capable of overcoming several of these long-standing obstacles in drug development.^4^ Ionic liquids are low-melting organic salts made up of bulky and asymmetric ions having adjustable polarity, high chemical and thermal stability, and low volatility.^5^ By altering cation–anion combinations, their physicochemical characteristics can be precisely controlled, enabling the logical development of solvent systems customized for particular drug molecules.^6–9^ ILs have multiple applications in the pharmaceutical industry, including the ability to function as stabilizers, solvents, co-solvents, and even bioactive ingredients in formulations. A paradigm shift away from traditional solid-state formulations and toward liquid or quasi-liquid therapeutic systems with improved pharmacological performance has resulted from their capacity to improve the solubility, permeability, and stability of drugs.^10–13^ Protic ionic liquids (PILs), which are formed by proton transfer between a Brønsted acid and a base, are particularly appealing among various IL classes because of their biocompatibility, capacity to form hydrogen bonds, and modifiable polarity. Strong and targeted interactions with pharmaceutical molecules are made possible by their dual hydrogen-bond donor–acceptor nature, which makes them attractive options for enhancing drug solvation and stabilization.^14–20^ PILs offer a molecular-level mechanism to improve drug delivery performance by efficiently modifying solute–solvent interactions, aggregation behavior, and redox stability.

Anthraquinones are aromatic compounds with a variety of biological activities, such as anticancer, antiproliferative, and antioxidant properties, and are a class of drugs that especially benefit from such solvation control.^20–28^ However, anthraquinone-based drugs frequently have low bioavailability and decreased therapeutic efficacy due to poor water solubility and aggregation. The polarity and hydrogen-bonding properties of their surroundings greatly influence their photophysical and redox behaviors.^29–36^ Protic ionic liquids provide a tunable medium that can improve the pharmacokinetic characteristics of anthraquinone derivatives by influencing solubility and molecular interactions through hydrogen bonding and electrostatic effects.^37–46^ A clinically used anthraquinone derivative, mitoxantrone (MTX), is a potent anticancer medication used to treat lymphoma, breast cancer, and leukemia. MTX has real-world formulation problems that make it hard to deliver effectively, even though it is more effective and less harmful to the heart than traditional anthracyclines like daunomycin and adriamycin. The pharmacological performance and therapeutic effectiveness of MTX can be improved by using ionic liquid-assisted strategies to overcome these problems. The present study investigates the intermolecular interactions between mitoxantrone (MTX) and three protic ionic liquids (PILs), ethanol ammonium acetate (EAAc), ethanol ammonium butyrate (EABu), and ethanol ammonium hexanoate (EAHx) in aqueous media. These PILs were selected because they share a common cationic structure, but with different anionic chain lengths, which enable the successive examination of the impact of anion size and hydrophobicity on the solvation and stabilization of MTX. These interactions were studied using a combination of spectroscopic, electrochemical and computational methods. Ultraviolet-visible and fluorescence spectroscopy have provided insight into the variations in the electronic and photophysical behavior of MTX, while cyclic voltammetry has shown redox stability changes in MTX due to the PILs. Density functional theory calculations also confirmed these experimental results, suggesting the thermodynamic and electronic factors controlling the interaction of MTX with PILs. Altogether, this work establishes the impact of protic ILs on the stability and binding of MTX. In this work, the binding of PIL with MTX has been examined, which has not been previously reported in the literature: although publications are available on imidazolium-based ILs, there have been no reports on a PIL. Herein, we report the interactions between MTX, a well-established standard drug, and PIL, along with the resulting structural changes. Computational studies have also been performed to support the experimental results.

## 2. Materials and methods

### 2.1 Materials

The reagents used were carefully selected and prepared to guarantee the reliability and reproducibility of the experiment. Acetic acid, butyric acid, hexanoic acid, TEMPO and MTX (commercial grade) were employed without further purification; ethanolamine was purchased separately. Before each experiment, chemicals were dried in a vacuum for 24 hours. Complete details of the chemicals used have been reported in Table 1.

**Table 1:** Chemicals and their specifications

Chemicals	CAS no.	Source	Purity^b^
			Mass fraction
Butyric acid	107-92-6	TCI Pvt. Ltd	≥0.99
Acetic acid	64-19-7	TCI Pvt. Ltd	≥0.99
Hexanoic acid	142-62-1	TCI Pvt. Ltd	≥0.99
Ethanolamine	141-43-5	Loba Chemie Pvt. Ltd	≥0.99
Ethanol ammonium acetate (EAAc)	54300-24-2	Synthesized	≥0.96^a^
Ethanol ammonium butyrate (EABu)	56409-18-8	Synthesized	≥0.96^a^
Ethanol ammonium hexanoate (EAHx)	—	Synthesized	≥0.96^a^
Mitoxantrone (MTX)	65271-80-9	TCI Pvt. Ltd	≥0.95

a Based on spectroscopic analysis (IR and NMR).

b As provided by the supplier.

#### 2.1.1 Preparation of stock solutions

Double-distilled water was used for the preparation of MTX, EAAc, EABu, and EAHx stock solutions to minimize ionic and particulate contaminations. This high purity is a prerequisite for the stability of the solution during the spectroscopic and electrochemical measurements. The stock solutions were kept at room temperature in amber glass containers to avoid light-induced degradation.

#### 2.1.2 Working solution concentrations

A solution of mitoxantrone (MTX, 20 µM) was formulated by diluting the stock solution with double-deionized water. This concentration was chosen to facilitate reliable spectroscopic measurements while still being interesting for interaction studies. Stock solutions of EAAc, EABu and EAHx were prepared at a concentration of 0.05 M to allow the comparison of their reactions with MTX. All solutions used were freshly prepared, then vortex-stirred and filtered with a 0.22 µm syringe filter to remove visible particles before spectroscopic and electrochemical experiments.

### 2.2 Synthesis and characterization of ethanol ammonium acetate (EAAc), ethanol ammonium butyrate (EABu), and ethanol ammonium hexanoate (EAHx) protic ionic liquid (PIL)

Protic ionic liquids (PILs) were synthesised *via* controlled acid–base neutralisation, following previously reported procedures.^12,29,46–52^ Briefly, ethanolamine was dissolved in methanol and placed in a double-neck round-bottom flask equipped with a dropping funnel. The respective acids for each PIL were added slowly: acetic acid for ethanol ammonium acetate (EAAc), butyric acid for ethanol ammonium butyrate (EABu), and hexanoic acid for ethanol ammonium hexanoate (EAHx). The neutralization reaction was performed at a low temperature of approximately 278.15 K to maintain reactivity control and minimize side reactions. To promote uniformity and efficient proton transfer, continuous magnetic stirring was maintained. To ensure complete reaction and PIL stabilisation, the resulting viscous liquid was stirred for 24 hours at room temperature (about 25 °C). The residual solvent and volatile impurities were removed by rotary evaporation carried out at 50 ± 2 °C for 4 hours under reduced pressure (∼200 mbar), followed by further drying in high vacuum (10^−3^ mbar) for more than 36 hours to remove trace moisture and residual amine. Karl Fischer titration (Metrohm KF 870 Titrino) showed that the moisture content was less than 200 ppm. This was taken into account when determining the molality of the aqueous PIL solutions. To confirm the synthesis of protic ionic liquids, deuterated dimethyl sulfoxide (DMSO-d6) served as the solvent in Fourier-transform infrared spectroscopy (FT-IR, PerkinElmer) and proton nuclear magnetic resonance spectroscopy (^1^H NMR, Bruker Avance 500 MHz). The FT-IR spectra showed a unique broad band around 1600 cm^−1^ that was made up of carbonyl ( <svg xmlns="http://www.w3.org/2000/svg" version="1.0" width="10.400000pt" height="16.000000pt" viewBox="0 0 10.400000 16.000000" preserveAspectRatio="xMidYMid meet"><metadata> Created by potrace 1.16, written by Peter Selinger 2001-2019 </metadata><g transform="translate(1.000000,15.000000) scale(0.011667,-0.011667)" fill="currentColor" stroke="none"><path d="M80 1160 l0 -40 40 0 40 0 0 -40 0 -40 40 0 40 0 0 -40 0 -40 40 0 40 0 0 -40 0 -40 40 0 40 0 0 -40 0 -40 40 0 40 0 0 -40 0 -40 40 0 40 0 0 -40 0 -40 40 0 40 0 0 80 0 80 -40 0 -40 0 0 40 0 40 -40 0 -40 0 0 40 0 40 -40 0 -40 0 0 40 0 40 -40 0 -40 0 0 40 0 40 -40 0 -40 0 0 40 0 40 -80 0 -80 0 0 -40z M560 520 l0 -40 -40 0 -40 0 0 -40 0 -40 -40 0 -40 0 0 -40 0 -40 -40 0 -40 0 0 -40 0 -40 -40 0 -40 0 0 -40 0 -40 -40 0 -40 0 0 -40 0 -40 -40 0 -40 0 0 -40 0 -40 80 0 80 0 0 40 0 40 40 0 40 0 0 40 0 40 40 0 40 0 0 40 0 40 40 0 40 0 0 40 0 40 40 0 40 0 0 40 0 40 40 0 40 0 0 80 0 80 -40 0 -40 0 0 -40z"/></g></svg> C=O) stretching and N–H plane bending vibrations. In addition, the 2400–3500 cm^−1^ range showed O–H stretches and unique N–H stretching vibrations of the ammonium groups. This aligns with our expectations for the PILs (Fig. S1–S3). The ^1^H NMR spectra and their peaks can be found in Fig. S4–S6 of the SI.

### 2.3 Absorption spectroscopy

A cutting-edge Shimadzu T80+ UV-visible spectrophotometer, equipped with high-precision quartz cuvette cells featuring a 1 cm path length, was used to conduct UV-visible spectroscopy and investigate the dynamic interactions between mitoxantrone (MTX) and protic ionic liquids (ILs). To examine the binding behavior of the three customized ionic liquids, ethanol ammonium acetate (EAAc), ethanol ammonium butyrate (EABu), and ethanol ammonium hexanoate (EAHx), absorption spectra of MTX were obtained both in their presence and absence. To create a precise baseline and guarantee measurement accuracy, double-distilled water was used as the blank. All spectroscopic measurements were performed in triplicate, and the spectra shown represent averaged data for reproducibility. UV-visible absorption spectra were recorded in the range of 190–700 nm. Stock solutions of mitoxantrone (MTX, 20 µM) and the protic ionic liquids EAAc, EABu, and EAHx were prepared with double-distilled water. In the absorbance studies, each ionic liquid was added systematically in a concentration range from 0.001 to 0.009 M in the presence of MTX, which was kept constant at a concentration of 20 µM. This approach allows for the examination of the effects of the ionic liquid concentration on the spectroscopic properties of MTX and provides useful information on the strength and nature of MTX–IL interactions.

### 2.4 Fluorescence emission spectroscopy

Fluorescence emission spectra were recorded to examine the interactions between mitoxantrone (MTX) and the protic ionic liquids ethanol ammonium acetate (EAAc), ethanol ammonium butyrate (EABu), and ethanol ammonium hexanoate (EAHx). The fluorescence experiments were carried out on an RF-5301 PC fluorescence spectrophotometer using a 1 cm quartz cuvette. Baseline corrections were made for double-distilled water as the blank. The excitation wavelength for MTX was fixed at 610 nm for all experiments, as mentioned in previous literature. Fresh stock solutions of MTX (20 µM) and PIL (0.05 M) were prepared in double-distilled water to avoid inconsistency and impurities. The concentrations of EAAc, EABu, and EAHx were varied systematically from 0.001 M to 0.007 M during fluorescence experiments. MTX concentration was kept constant at 20 µM while varying the ionic liquid concentration to study the effects of the ionic liquids on the fluorescence behavior of MTX. The binding interactions were quantified by evaluating important parameters such as the binding constant (*K*_b_) and the Stern–Volmer constant (*K*_sv_). This experimental setup allowed for a proper determination of the strength and nature of MTX–IL interactions and provided molecular-level information useful in the design of ionic-liquid-based drug-delivery systems.

### 2.5 Cyclic voltammetry

Cyclic voltammograms were recorded with a Metrohm Autolab PGSTAT302N potentiostat to study the redox activity of mitoxantrone (MTX) and its stability in the presence of protic ionic liquids (PILs). Cyclic voltammetry measurements were conducted at 25 °C in a standard three-electrode system using a glassy carbon working electrode, a platinum counter electrode, and an Ag/AgCl reference electrode. Before each measurement, the glassy carbon electrode was cleaned with double-distilled water and carefully wiped dry using lint-free tissue to achieve a clean, reproducible electrode surface.

#### 2.5.1 Solution preparation for cyclic voltammetry

Electrochemical studies were performed with mitoxantrone (MTX, 20 µM) dissolved in a supporting electrolyte consisting of potassium nitrate (KNO_3_) and 2,2,6,6-tetramethyl-1-piperidinyloxy (TEMPO). KNO_3_ was used for its ionic conductivity, while TEMPO acted as a redox mediator to facilitate the electrochemical response. Solutions of ethanol ammonium acetate (EAAc), ethanol ammonium butyrate (EABu), and ethanol ammonium hexanoate (EAHx) were prepared at 0.05 M in the same support electrolyte to maintain consistency. The binding between MTX and the protic ionic liquids was studied by performing cyclic voltammetry using a fixed MTX concentration of 20 µM in various concentrations of EAAc or EABu (0.001–0.009 M). The goal of this study was to determine the redox behavior of MTX and the thermodynamic stability of MTX-PILs complexes. Despite the relatively low concentrations employed, the ionic nature of the PILs promotes strong electrostatic and hydrogen-bonding interactions with MTX, which significantly influence its electrochemical response and stabilisation in solution.

### 2.6 Density functional theory

Computational analysis was performed to investigate the molecular properties and interactions of mitoxantrone (MTX) with protic ionic liquids (ILs). Molecular structures were visualised using GaussView 6.0, and density functional theory (DFT) calculations were carried out with Gaussian 16 W. Geometry optimisations were performed for MTX, ethanol ammonium acetate (EAAc), ethanol ammonium butyrate (EABu), ethanol ammonium hexanoate (EAHx), and their corresponding MTX–IL complexes (MTX + EAAc, MTX + EABu, and MTX + EAHx). All calculations employed the B3LYP hybrid functional with the 6-31G basis set, providing a balanced description of the electronic structure, stability, and interaction energetics of the systems studied.

## 3. Results and discussion

### 3.1. Analysis of the UV-visible spectrum

**Fig. 1:**
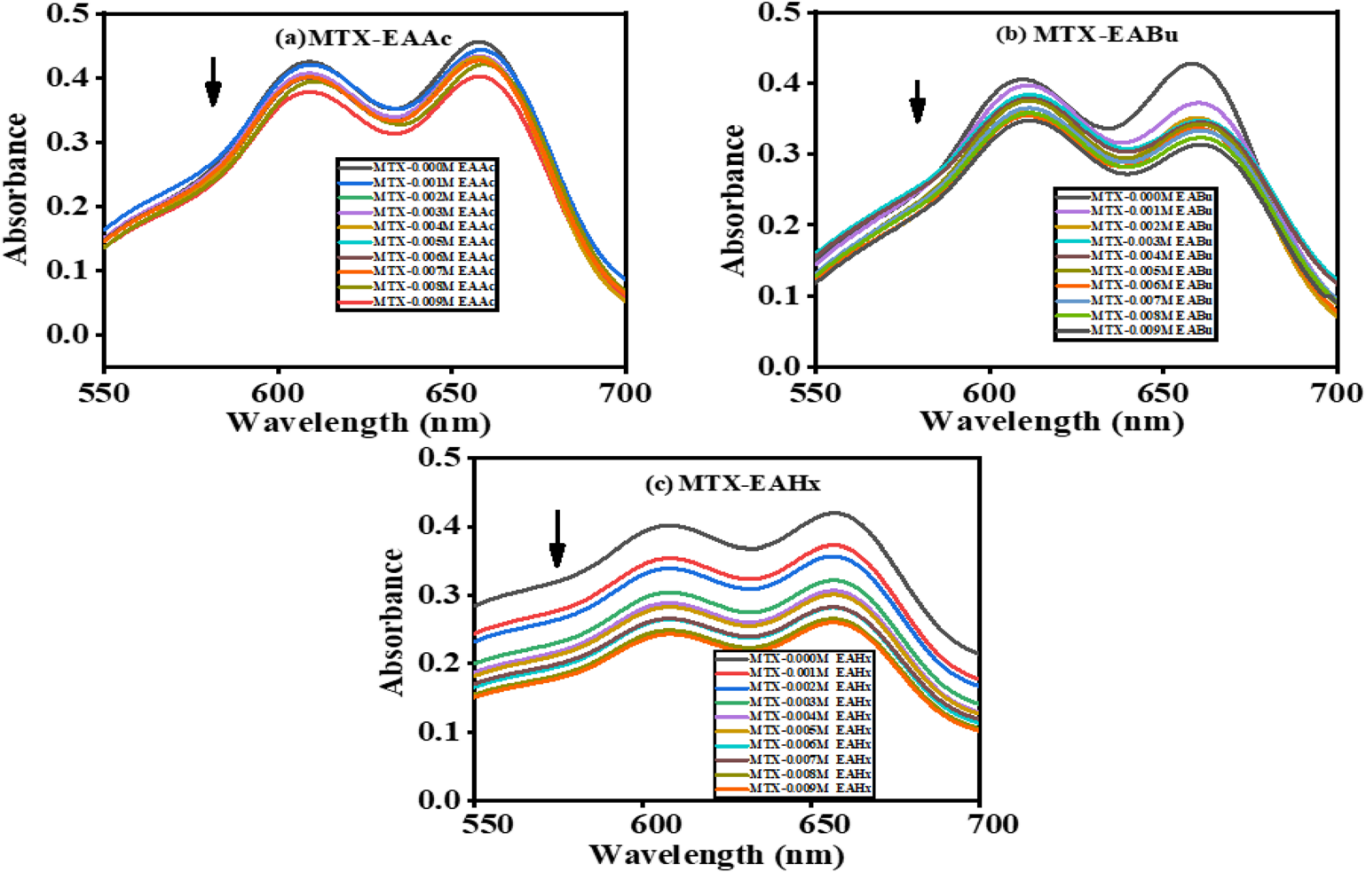
UV-visible spectra of mitoxantrone (MTX) (20 µM) with the increasing concentrations of (a) ethanol ammonium acetate (EAAc), (b) ethanol ammonium butyrate (EABu), and (c) ethanol ammonium hexanoate (EAHx).

The interactions between the drug and ionic liquids (ILs) have been studied using the UV-visible absorption technique. The relationships between MTX and the ILs EAAc, EABu, and EAHx have been the subject of several experiments, as shown in Fig. 1.

In an aqueous medium, mitoxantrone (MTX) exhibits two different absorption bands at *λ*_abs_ = 608 nm and *λ*_abs_ = 658 nm.^52^ The conjugated anthraquinone ring system is involved in π–π* transitions, which are responsible for the strong peak at 658 nm. The n–π* transitions, which involve non-bonding electrons on hydroxyl and amino groups, are responsible for the peak at 608 nm.^31–33^ The UV-visible absorption spectra of mitoxantrone (MTX) (20 µM) with varying concentrations of ionic liquids (ILs), ethanol ammonium acetate (EAAc), ethanol ammonium butyrate (EABu), and ethanol ammonium hexanoate (EAHx) (0.001–0.009 µM) have been recorded in water. With an increase in the concentrations of the ionic liquids (ILs), ethanol ammonium acetate (EAAc), ethanol ammonium butyrate (EABu), and ethanol ammonium hexanoate (EAHx), there was a decrease in the intensity of the absorbance of the mitoxantrone (MTX) peak at 658 nm (hypochromic shift) as shown in Fig. 1. Eqn (1) and (2) have been used to determine the binding constant (*K*_b_) and free energy of binding (Δ*G*_b_):1
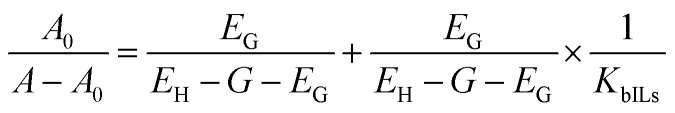
where *A*_0_, *ε*_G_, *A*, and *ε*_H–G_ are the absorbance and absorbance coefficients of MTX in the absence and presence of IL, respectively.

2 Δ*G* = −*RT* ln *K*_b_

**Fig. 2:**
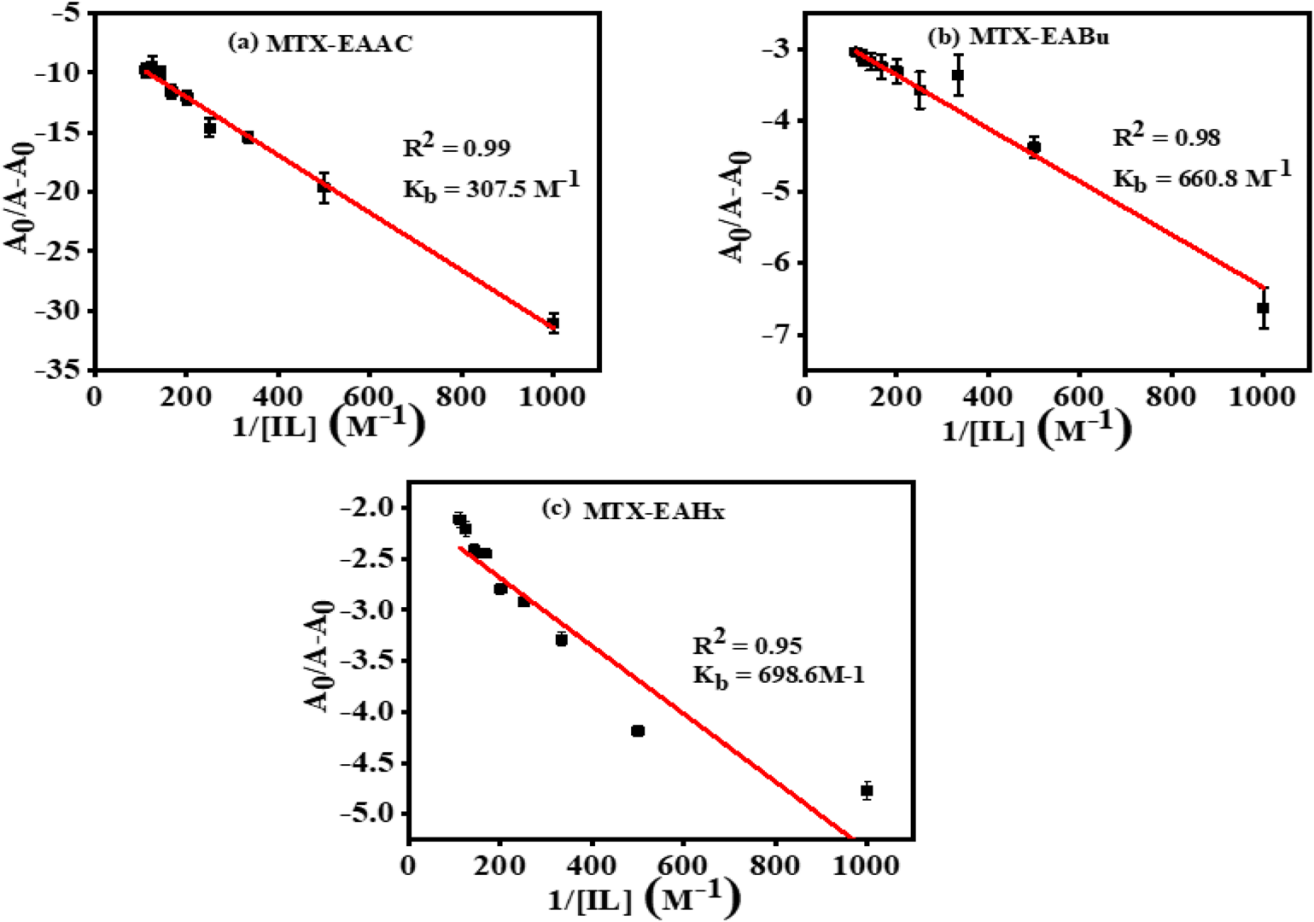
Plots of *A*_0_/*A* − *A*_0_*vs.* 1/[ILs] of (a) mitoxantrone + ethanol ammonium acetate (MTX + EAAc), (b) mitoxantrone + ethanol ammonium butyrate (MTX + EABu), and (c) mitoxantrone + ethanol ammonium hexanoate (MTX + EAHx).

Here, *R* = 8.314 J K^−1^ mol^−1^ and *T* = 298 K. The plot of 
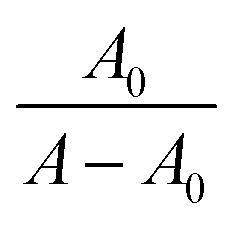
*vs.*
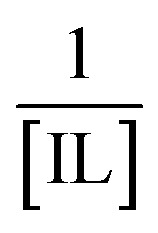
 is linear, and the binding constant (*K*_b_) has been calculated from the ratio of intercept to slope, as shown in Fig. 2. At 298.15 K, the binding constant (*K*_b_) values of the mitoxantrone (MTX) complexes with ethanol ammonium acetate (EAAc), ethanol ammonium butyrate (EABu), and ethanol ammonium hexanoate (EAHx) were determined to be 307.51 M^−1^, 660.86 M^−1^, and 698.67 M^−1^, respectively. The observed rise in *K*_b_ with the length of the anion chain (acetate < butyrate < hexanoate) suggests that the MTX–IL complexes are stabilized primarily by hydrophobic contacts. Compared to acetate and butyrate, the hexanoate anion (CH_3_CH_2_CH_2_CH_2_CH_2_COO^−^) has a longer alkyl chain, which improves hydrophobic and van der Waals interactions with the aromatic sections of MTX. Because the hydrophobic chains aid in keeping water molecules out of the binding interface and lowering the system's free energy, these interactions result in stronger complex formation and better binding affinity. Furthermore, the oxygen and nitrogen atoms of MTX (such as carbonyl or hydroxyl groups) can interact electrostatically or establish hydrogen bonds with the ethanol ammonium cation (NH_4_^+^) in these ionic liquids. Better spatial accommodation and interaction with MTX are made possible by the longer and more flexible hexanoate anion in EAHx, which further stabilizes the complex. Therefore, the higher (*K*_b_) value and greater stability of MTX–EAHx are due to both hydrophobic and electrostatic interactions. Calculated binding parameters have been compared with the literature data available^33^ and are shown in Table 2.

**Table 2:** *K*_b_, *R*^2^, and Δ*G* values calculated using UV-visible spectroscopy, fluorescence spectroscopy, and cyclic voltammetry for the mitoxantrone + ethanol ammonium acetate (MTX + EAAc), mitoxantrone + ethanol ammonium butyrate (MTX + EABu), and mitoxantrone + ethanol ammonium hexanoate (MTX + EAHx) systems

Techniques	Systems	Literature	*K*_b_ (L mol^−1^)	*R* ^2^	Δ*G* (kJ mol^−1^)
UV-visible	Mitoxantrone + ethanol ammonium acetate (MTX + EAAc)	FLZ-[C_10_mim][Cl]	3.07 × 10^2^ (expt.)	0.99	−14.17 (expt.)
			4.05 × 10^4^ (ref. 33)		−26.28 (ref. 33)
	Mitoxantrone + ethanol ammonium butyrate (MTX + EABu)	FLZ-[C_6_mim][Cl]	6.60 × 10^2^ (expt.)	0.98	−16.08 (expt.)
			6.78 × 10^3^ (ref. 33)		−24.32 (ref. 33)
Fluorescence	Mitoxantrone + ethanol ammonium acetate (MTX + EAAc)	FLZ-[C_10_mim][Cl]	3.31 (expt.)	0.97	−2.96 (expt.)
		FLZ-[C_6_mim][Cl]	3.30 × 10^4^ (ref. 33)		−25.65 (ref. 33)
			1.45 × 10^4^ (ref. 33)		−23.74 (ref. 33)
	Mitoxantrone + ethanol ammonium butyrate (MTX + EABu)	CIP-[C_10_mim][BF_4_]	5.62 (expt.)	0.96	−4.28 (expt.)
		CIP-[C_10_mim][Br]	48.62 (ref. 56)		−9.62 (ref. 56)
		CIP-[C_10_mim][Cl]	33.11 (ref. 56)		−8.67 (ref. 56)
			25.56 (ref. 56)		−8.03 (ref. 56)
Cyclic voltammetry	Mitoxantrone + ethanol ammonium acetate (MTX + EAAc)	DH + C_14_mimBr	5.57 × 10^2^ (expt.)	0.98	−15.67 (expt.)
		DH + TTAB	26.54 × 10^4^ (ref. 35)		−30.95 (ref. 35)
		AC + C_14_mimBr	7.45 × 10^4^ (ref. 35)		−27.81 (ref. 35)
		AC + TTAB	0.43 × 10^4^ (ref. 35)		−20.73 (ref. 35)
			0.19 × 10^4^ (ref. 35)		−18.70 (ref. 35)
	Mitoxantrone + ethanol ammonium butyrate (MTX + EABu)	LVF-[Bmim][Cl]	7.61 × 10^2^ (expt.)	0.95	−16.44 (expt.)
		LVF-[Dmim][Cl]	3.70 (ref. 30)		−3.24 (ref. 30)
			34.50 (ref. 30)		−8.54 (ref. 30)

Because of the bigger, bulkier hexanoate ion, the interaction between mitoxantrone (MTX) and ethanol ammonium hexanoate (EAHx) (698.67 M^−1^) may entail more substantial non-covalent interactions (*e.g.*, hydrophobic, electrostatic, and π–π stacking), which could result in stronger complexation. The smaller and more polar butyrate and acetate anions of ethanol ammonium butyrate (EABu) (660.86 M^−1^) and ethanol ammonium acetate (EAAc) (307.51 M^−1^), on the other hand, are likely to result in weaker interactions, which are reflected in smaller binding constants and less obvious spectral shifts. The free energy of binding (Δ*G*) was calculated to be −14.1 kJ mol^−1^ for mitoxantrone + ethanol ammonium acetate (MTX + EAAc), −16.1 kJ mol^−1^ for mitoxantrone + ethanol ammonium butyrate (MTX + EABu), and −16.3 kJ mol^−1^ for mitoxantrone + ethanol ammonium hexanoate (MTX + EAHx) using eqn (2). The process was spontaneous, as evidenced by the negative value of the change in the free energy of binding. The greater binding seen with EAHx suggests that this complex can provide a more stable and lipophilic drug–ionic liquid combination from the standpoint of drug delivery. Drug encapsulation, membrane permeability, and prolonged release all benefit from such increased lipophilicity. Longer hydrophobic chains in ionic liquids are known to improve the drug's bioavailability and controlled release characteristics by making it more compatible with lipid-based carriers (such as liposomes, micelles, or nanoparticles). The MTX–EAHx complex is therefore a viable system for targeted and long-term drug delivery applications because of its greater binding constant, which may result in enhanced stability, regulated release, and effective cellular uptake.

### 3.2. Fluorescence spectral studies

**Fig. 3:**
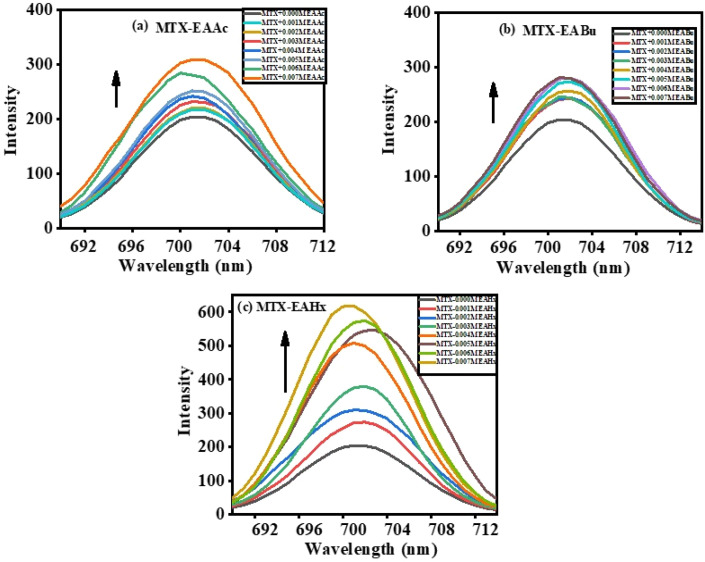
Fluorescence spectra of mitoxantrone (MTX) (20 µM) with the increasing concentrations of (a) ethanol ammonium acetate (EAAc), (b) ethanol ammonium butyrate (EABu), and (c) ethanol ammonium hexanoate (EAHx).

Mitoxantrone (MTX) exhibits a fluorescence peak at approximately 702 nm when exposed at 610 nm (Fig. 3). The addition of the ionic liquids (ILs), namely, ethanol ammonium acetate (EAAc), ethanol ammonium butyrate (EABu), and ethanol ammonium hexanoate (EAHx), enhanced mitoxantrone's fluorescence emission intensity. The complex formation between ionic liquids (ILs), ethanol ammonium acetate (EAAc), ethanol ammonium butyrate (EABu), ethanol ammonium hexanoate (EAHx), and mitoxantrone (MTX) was indicated by the rise in the emission spectra of mitoxantrone (MTX) upon the addition of ionic liquids (ILs),^53–55^ as represented in Fig. 3.

A gradual increase in fluorescence intensity was observed upon the addition of ionic liquid, indicating binding-induced fluorescence enhancement. The relative fluorescence intensity (*F*/*F*_0_) increased in a concentration-dependent manner. These plots show the concentration-dependent enhancement, and the corresponding variations in the fluorescence emission spectra were investigated.

**Fig. 4:**
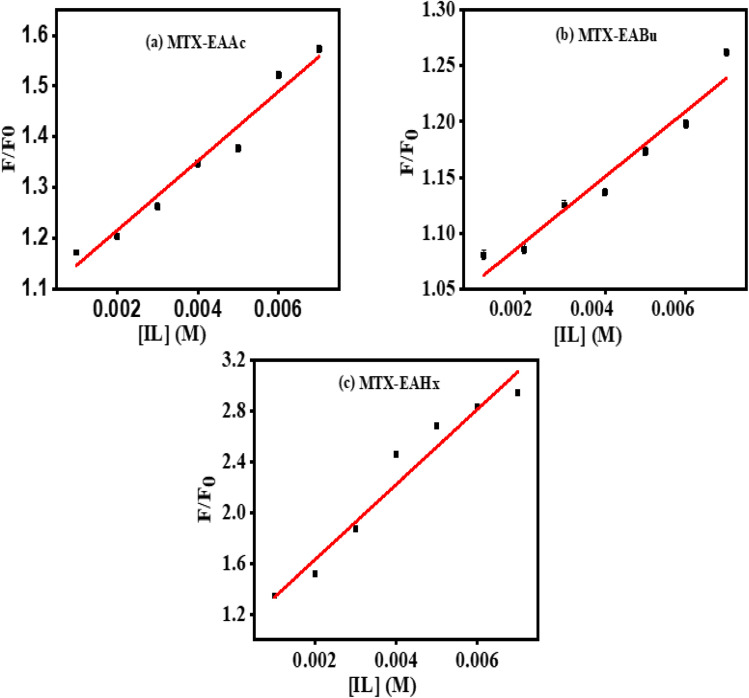
Plots of *F*/*F*_0_*vs.* [ILs] of (a) mitoxantrone + ethanol ammonium acetate (MTX + EAAc), (b) mitoxantrone + ethanol ammonium butyrate (MTX + EABu), and (c) mitoxantrone + ethanol ammonium hexanoate (MTX + EAHx).

*F*/*F*_0_ plots of (MTX + EAAc), (MTX + EABu), and (MTX + EAHx) complexes against the concentration of ILs are presented in Fig. 4.

**Fig. 5:**
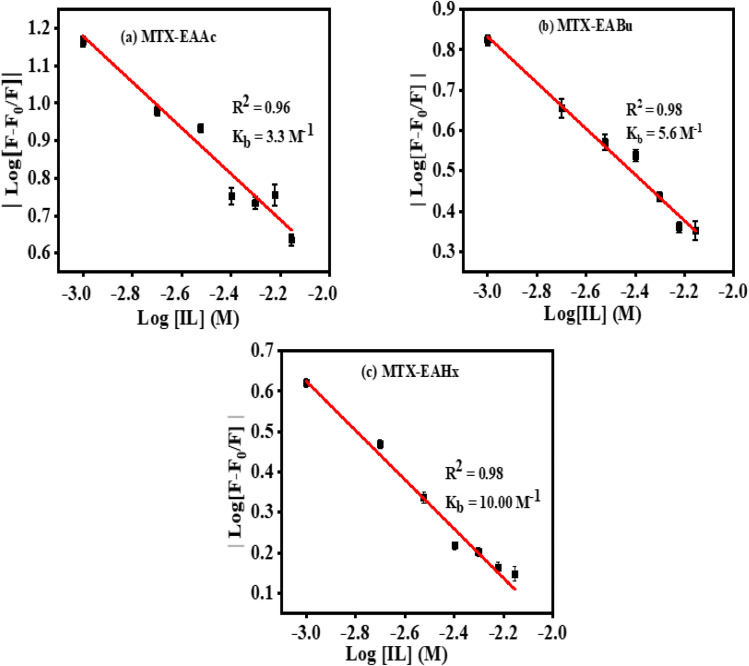
Plots of log *F* − *F*_0_/*F vs.* log[ILs] of (a) mitoxantrone + ethanol ammonium acetate (MTX + EAAc), (b) mitoxantrone + ethanol ammonium butyrate (MTX + EABu), and (c) mitoxantrone + ethanol ammonium hexanoate (MTX + EAHx).

*K*_b_ was calculated using eqn (3):3
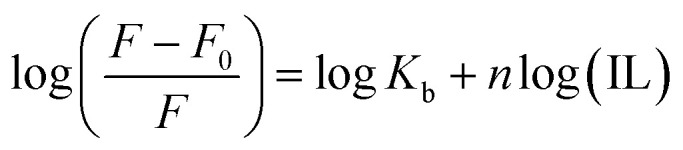
where *K*_b_ is the binding constant, and *n* is the number of binding sites. The linear fitting of the 
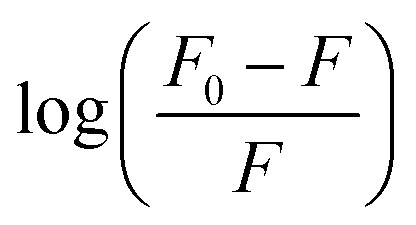
*vs.* log(IL) plot is presented in Fig. 5, where the slope provides the number of binding sites (*n*), and the intercept can be used to determine the binding constant (*K*_b_). EAHx had a higher *K*_b_ (10.00 M^−1^) value than EABu (5.62 M^−1^) and EAAc (3.31 M^−1^), indicating a greater binding affinity of EAHx with MTX as compared to EABu and EAAc. Compared to EABu (butyrate) and EAAc (acetate), EAHx has a longer alkyl chain (hexanoate). The extended chain improves molecular association by increasing hydrophobic contacts with MTX's aromatic sections. By stabilizing the MTX–EAHx complex, these hydrophobic forces increase *K*_b_. The large hexanoate anion strengthens binding by offering a larger surface area for van der Waals and potential π–π stacking interactions with the planar anthracenedione rings of MTX. Because EAHx is more hydrophobic, it tends to keep water molecules out of the MTX binding area, which lessens solvent interference and promotes a stronger drug–IL connection. The values of Δ*G* for the complexation between (MTX) and ILs have been calculated using eqn (4).

4 Δ*G* = −*RT* ln *K*_b_

The negative value of (Δ*G*) indicates the spontaneous complex formation process between the ionic liquids (ILs), ethanol ammonium acetate (EAAc), ethanol ammonium butyrate (EABu) and ethanol ammonium hexanoate (EAHx), and mitoxantrone (MTX). The mitoxantrone + ethanol ammonium hexanoate (MTX + EAHx) complex (−10.00 kJ mol^−1^) is more favorable than the mitoxantrone + ethanol ammonium butyrate (MTX + EABu) (−4.28 kJ mol^−1^) and mitoxantrone + ethanol ammonium acetate (MTX + EAAc) (−2.96 kJ mol^−1^) complexes due to the lower (Δ*G*) values. It can be concluded that the MTX-IL complex formation is largely influenced by hydrophobic and electrostatic forces. A stronger interaction between mitoxantrone and the longer-chain ionic liquid, ethanol ammonium hexanoate, is shown by the higher binding constant (*K*_b_) of the MTX–EAHx complex (10.00 M^−1^) in comparison to MTX–EABu (5.62 M^−1^), and MTX–EAAc (3.31 M^−1^). This greater binding implies that MTX is better stabilized and encapsulated in the EAHx environment, preventing degradation and increasing its solubility. Such moderate-to-strong binding is advantageous for producing controlled or sustained drug release in the context of drug delivery because it permits progressive release at the target site while maintaining the drug's stability throughout transport. Therefore, in comparison to the shorter-chain equivalents, EABu and EAAc, EAHx seems to offer an ideal milieu for efficient MTX administration. Therefore, the fluorescence data indicate that EAHx provides a better milieu for MTX binding and stabilization than EAAc or EABu, making it a feasible ionic liquid for MTX-based drug delivery systems. The computed binding parameters, as displayed in Table 2, have been compared with the data from the literature.^30,33,56^

### 3.3. Cyclic voltammetry studies

Mitoxantrone (MTX), a synthetic anthracenedione anticancer drug, exhibits tautomeric flexibility due to proton and electron redistribution within its quinone core and aminoalkyl side chains. This structural adaptability plays an important role in governing the chemical reactivity and electrochemical behaviour of the molecule.^57,58^ In cyclic voltammetry (CV), MTX displays a well-defined reversible oxidation peak at lower positive potentials, which is attributed to the oxidation of the 5,8-hydroxyl groups on the anthracenedione framework, indicating a stable and thermodynamically favourable redox process. In addition to this primary response, a second oxidation peak is observed at more positive potentials. Previous studies have associated this feature with the presence of a less abundant electroactive tautomer formed through intramolecular rearrangement involving the side-chain nitrogen and the quinone system. Due to its different electronic structure, this tautomer has a higher oxidation potential and hence gives the secondary voltammetric signal. The influence of the solvent medium on this tautomeric equilibrium was explored by performing cyclic voltammetry measurements in the presence of protic ionic liquids (PILs), namely ethanol, ammonium acetate, butyrate, and hexanoate. Due to their marked hydrogen-bonding capability and ionic nature, PILs strongly alter the solvation environment of MTX, especially at heteroatoms involved in the proton-transfer process. Notably, CV experiments conducted in ethanol ammonium acetate showed a pronounced decrease in the intensity of the second oxidation peak, suggesting suppression of the electroactive tautomer. These observations demonstrate that PILs can modulate the tautomeric distribution of MTX and, consequently, its redox behaviour, highlighting the sensitivity of MTX electrochemistry to solvent–solute interactions.^59,60^

**Fig. 6:**
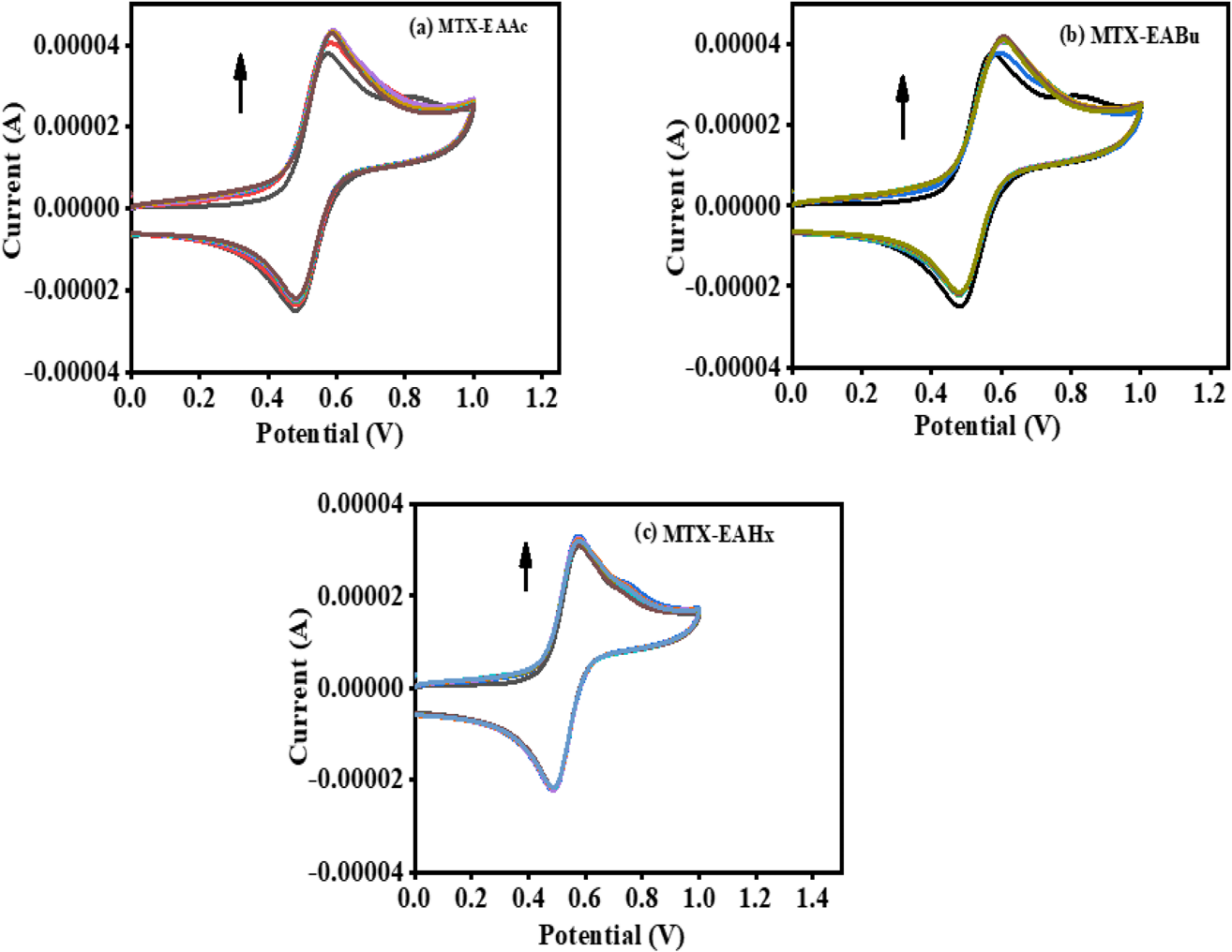
Cyclic voltammograms of mitoxantrone (MTX) (20 µM) as a function of the increasing concentrations of (a) ethanol ammonium acetate (EAAc), (b) ethanol ammonium butyrate (EABu), and (c) ethanol ammonium hexanoate (EAHx).

Cyclic voltammograms of pure mitoxantrone (MTX), and mitoxantrone (MTX) with varied concentrations of ethanol ammonium acetate (EAAc) and ethanol ammonium butyrate (EABu), ethanol ammonium hexanoate (EAHx), are shown in Fig. 6. It was found that when ethanol ammonium acetate (EAAc), ethanol ammonium butyrate (EABu), and ethanol ammonium hexanoate (EAHx) concentrations are added incrementally, the peak current (*i*_p_) of mitoxantrone (MTX) increases. The development of the electrochemically active mitoxantrone (MTX) and ionic liquids (ILs) complex may be the cause of this notable rise in peak current values.

**Fig. 7:**
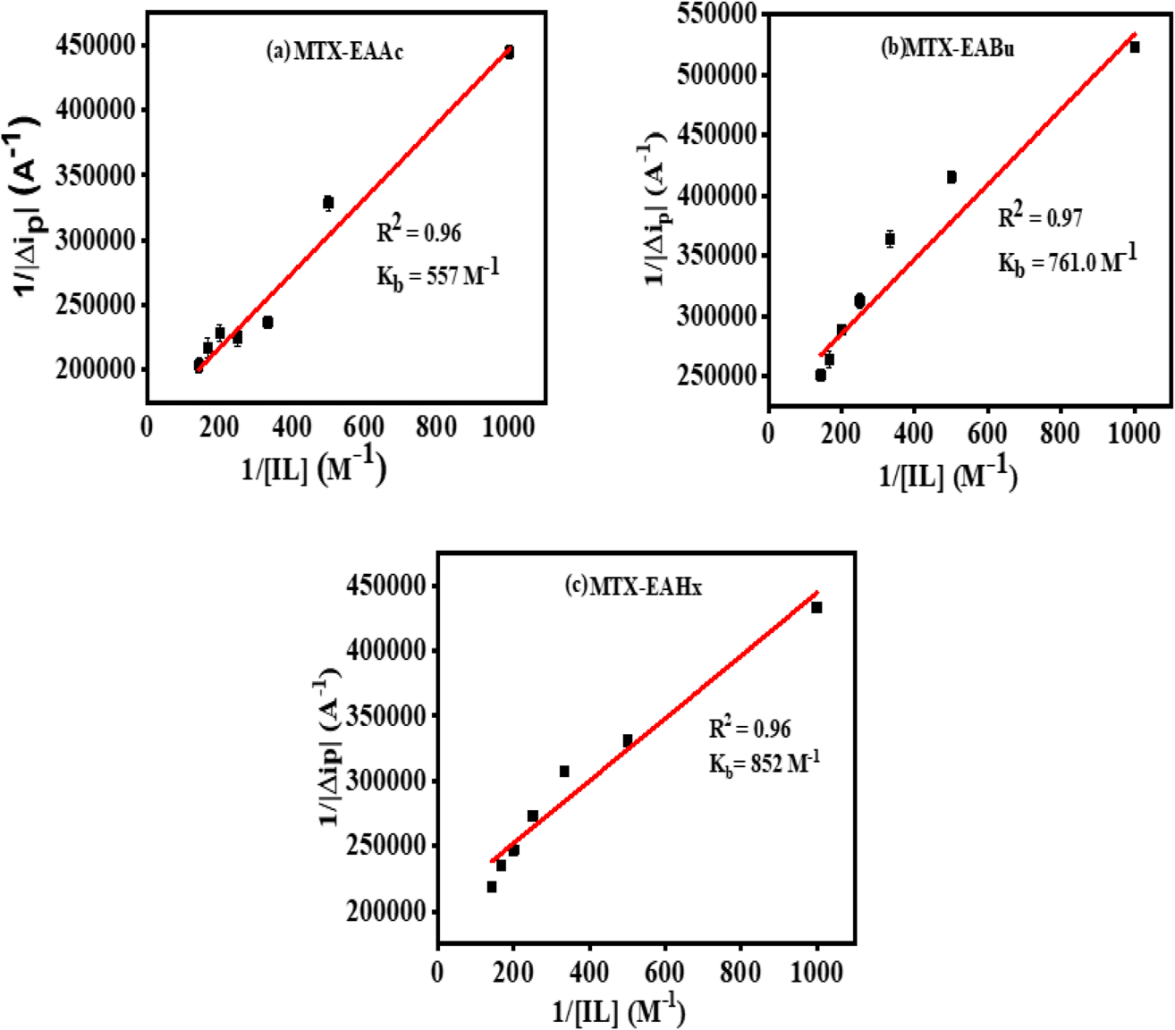
Plots showing the linear relationship between 
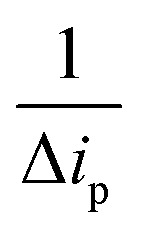
 and 
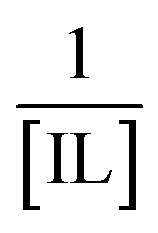
 for mitoxantrone (MTX) (20 µM) with increasing concentrations of (a) ethanol ammonium acetate (EAAc), (b) ethanol ammonium butyrate (EABu), and (c) ethanol ammonium hexanoate (EAHx) complexes.

According to the Langmuir eqn (5), the increase in the peak current value (Δ*i*_p_) of mitoxantrone (MTX) in the presence of different concentrations of ionic liquids, namely, ethanol ammonium acetate (EAAc), ethanol ammonium butyrate (EABu), and ethanol ammonium hexanoate (EAHx), indicates a good linear relationship between the reciprocal of peak current and concentration:5
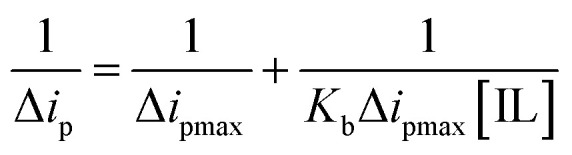
where Δ*i*_p_ = *i*_0_ − *i*, *i*_0_ and *i* are the peak currents of mitoxantrone (MTX) in the absence and presence of ionic liquid; *K*_b_ = binding constant; and [IL] = concentration of the ionic liquids (ethanol ammonium acetate (EAAc)), ethanol ammonium butyrate (EABu), and ethanol ammonium hexanoate (EAHx)). The ratio of the intercept and slope of the 1/Δ*i*_p_*versus* 1/[IL] plot gives the value of the binding constant (*K*_b_), as shown in Fig. 7. The higher binding constant obtained for ethanol ammonium hexanoate (EAHx)) (*K*_b_ = 852.0 L mol^−1^) than ethanol ammonium butyrate (EABu) (*K*_b_ = 761.085 L mol^−1^) and ethanol ammonium acetate (EAAc) suggests the favorable binding of mitoxantrone (MTX) with ethanol ammonium hexanoate (EAHx) over ethanol ammonium butyrate (EABu) and ethanol ammonium acetate (EAAc) (Table 1). The mitoxantrone + ethanol ammonium hexanoate (MTX + EAHx) complex (−16.72 kJ mol^−1^) is more favorable than the mitoxantrone + ethanol ammonium butyrate (MTX + EABu) complex (−16.44 kJ mol^−1^) and the mitoxantrone + ethanol ammonium acetate (MTX + EAAc) complex (−15.67 kJ mol^−1^) due to a low Δ*G* value. The calculated binding parameters have been compared with available literature data,^30,35^ as shown in Table 2. In ethanol ammonium hexanoate, the hexanoate ion may interact with mitoxantrone more strongly than the butyrate ion and the acetate ion through hydrogen bonds or electrostatic forces. The hexanoate ion likely adopts a more favorable spatial arrangement because of its longer alkyl chain, resulting in enhanced stabilization of the complex. The hexanoate group may offer a steric shape that is better for complexation with mitoxantrone than the smaller butyrate and acetate groups. The larger size of the hexanoate may lead to improved spatial interaction with the drug molecule. This can shift the potential or alter the peak current in the cyclic voltammetry scan. Ethanol ammonium hexanoate (EAHx) alters the solvation and dielectric environment of mitoxantrone (MTX), leading to measurable changes in peak current and peak shape in cyclic voltammograms. Stronger MTX–EAHx interactions enhance drug stabilisation within the ionic liquid matrix, enabling more controlled and sustained behaviour compared to shorter-chain ionic liquids. These results provide electrochemical support for ionic-liquid-assisted strategies to improve MTX stability and delivery.

### 3.4. Density functional theory

#### 3.4.1 Frontier molecular orbital (MO) analysis

**Fig. 8:**
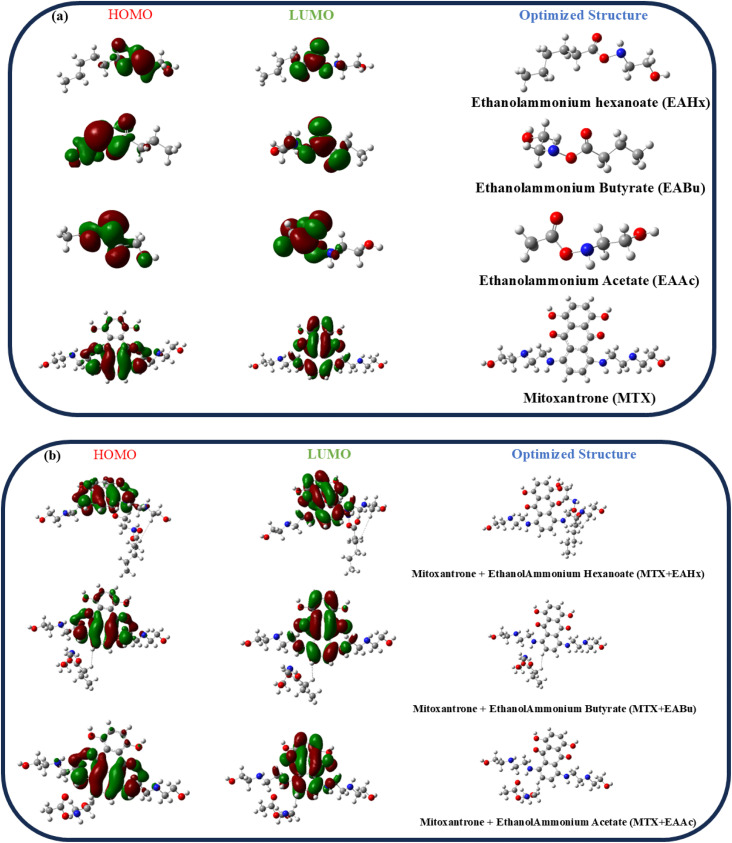
Optimized structures and frontier molecular orbitals (HOMO–LUMO) of (a) mitoxantrone (MTX), ethanol ammonium acetate (EAAc), ethanol ammonium butyrate (EABu), and ethanol ammonium hexanoate (EAHx). (b) Frontier molecular orbitals (HOMO–LUMO) of mitoxantrone + ethanol ammonium acetate (MTX + EAAc), mitoxantrone + ethanol ammonium butyrate (MTX + EABu), and mitoxantrone ethanol ammonium hexanoate (MTX + EAHx) complexes.

The optimized structures of mitoxantrone (MTX), ethanol ammonium acetate (EAAc), ethanol ammonium butyrate (EABu), ethanol ammonium hexanoate (EAHx), mitoxantrone + ethanol ammonium acetate (MTX + EAAc), and mitoxantrone + ethanol ammonium butyrate (MTX + EABu), mitoxantrone + ethanol ammonium hexanoate (MTX + EAHx) are shown in Fig. 8, together with the lowest unoccupied molecular orbital (LUMO) and highest occupied molecular orbital (HOMO) structures.

**Fig. 9:**
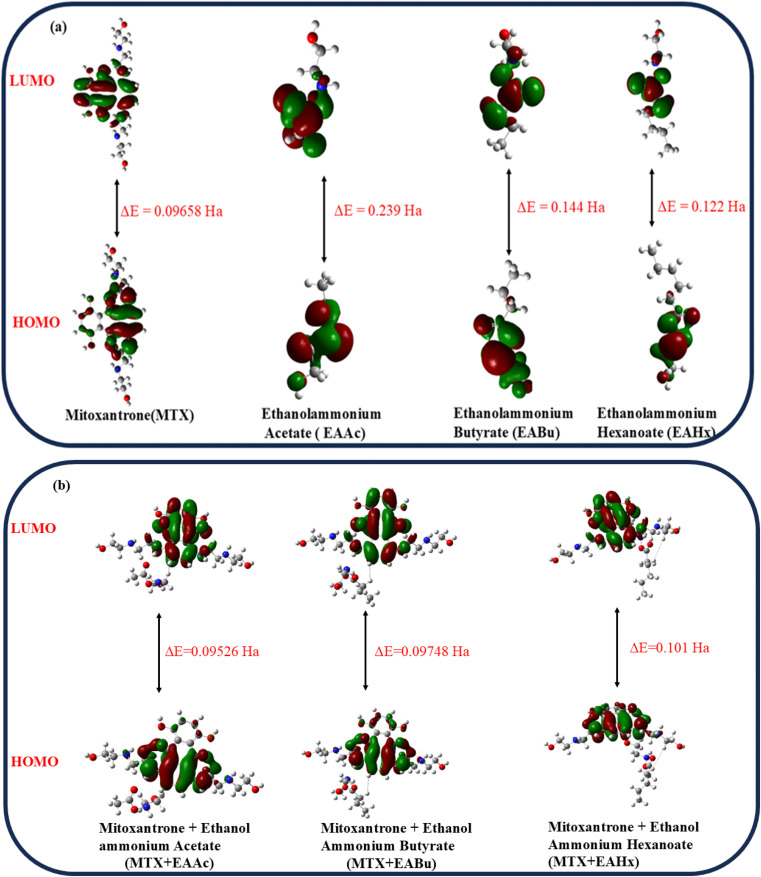
HOMO–LUMO energy gaps of (a) mitoxantrone (MTX), ethanol ammonium acetate (EAAc), ethanol ammonium butyrate (EABu), and ethanol ammonium hexanoate (EAHx). (b) HOMO–LUMO energy gaps of mitoxantrone + ethanol ammonium acetate (MTX + EAAc), mitoxantrone + ethanol ammonium butyrate (MTX + EABu), and mitoxantrone ethanol ammonium hexanoate (MTX + EAHx).

To determine the strength of contacts in the MTX + IL complexes and the viability of electron transfer between the drug and IL donor–acceptor, the energy difference (Δ*E*) has been calculated (Fig. 9). The calculated HOMO–LUMO energy gaps for mitoxantrone (MTX), ethanol ammonium acetate (EAAc), ethanol ammonium butyrate (EABu), ethanol ammonium hexanoate (EAHx), mitoxantrone + ethanol ammonium acetate (MTX + EAAc), mitoxantrone + ethanol ammonium butyrate (MTX + EABu), and mitoxantrone + ethanol ammonium hexanoate (MTX + EAHx) are 0.09658, 0.239, 0.144, 0.125, 0.09526, 0.09748, and 0.101 Ha, respectively.

#### 3.4.2 Characteristics of quantum chemistry

Quantum chemical descriptors derived from DFT calculations provide valuable insight into the electronic structure, stability, and reactivity of the mitoxantrone (MTX)–ionic liquid complexes. Parameters such as ionization potential (*I*), electron affinity (*A*), electronegativity (*χ*), hardness (*η*), softness (*S*), and electrophilicity index (*ω*) were analysed to elucidate the nature of interactions between MTX and ethanol ammonium acetate (EAAc), butyrate (EABu), and hexanoate (EAHx). These descriptors explain the experimentally observed stability and binding trends by relating them to fundamental electronic effects. From Table 3, it can be seen that electron affinity decreases in the order of MTX + EAAc > MTX + EABu > MTX + EAHx, which shows that MTX + EAAc has a greater tendency to accept electrons and thus exhibits a higher chemical reactivity. The lower electron affinity of MTX + EAHx means lower reactivity and higher stability. A similar trend is seen in electronegativity, which decreased from MTX + EAAc to MTX + EAHx. Lower electronegativity values indicate a diminished tendency to attract electron density, consistent with increased resistance to electronic perturbation. Together, the reduced electron affinity and electronegativity of the MTX + EAHx complex point to greater electronic stability and lower reactivity when compared to the MTX + EABu and MTX + EAAc systems. These findings support the conclusion that increasing the alkyl chain length in the ionic liquid anion enhances the stabilisation of MTX, in agreement with experimental spectroscopic and electrochemical observations. As a result, their electrical structure is stable since they are less prone to giving or receiving electrons easily. Hard molecules generally participate less in processes that require significant distortion or polarization of the electron cloud. Hardness is a measure of a molecule's resistance to change in its electronic structure. A molecule with a greater hardness value is less reactive and more stable. The mitoxantrone + ethanol ammonium acetate (MTX + EAAc) complex shows a lower hardness value (0.04763 Ha) as compared to the mitoxantrone + ethanol ammonium butyrate (MTX + EABu) complex (0.048 Ha) and the mitoxantrone + ethanol ammonium hexanoate (MTX + EAHx) complex (0.050 Ha). This indicates that upon complexation, mitoxantrone + ethanol ammonium hexanoate (MTX + EAHx) becomes harder and more stable as compared to mitoxantrone + ethanol ammonium butyrate (MTX + EABu) and mitoxantrone + ethanol ammonium acetate (MTX + EAAc) complexes. Similarly, a higher softness value means a more reactive material. The mitoxantrone + ethanol ammonium acetate (MTX + EAAc) complex shows a slightly higher softness value (10.49 Ha^−1^) as compared to the mitoxantrone + ethanol ammonium butyrate (MTX + EABu) complex (10.25 Ha^−1^) and the mitoxantrone + ethanol ammonium hexanoate (MTX + EAHx) complex (9.90 Ha^−1^), as shown in Table 3. The following physicochemical descriptors were calculated from eqn (7)–(13): chemical hardness (*η*), softness (*S*), electrophilicity index (*ω*), electronegativity (*χ*), ionization potential (IP), chemical potential (*µ*), and electron affinity (EA).

7 *η* = (*E*_LUMO_ − *E*_HOMO_)/2

8
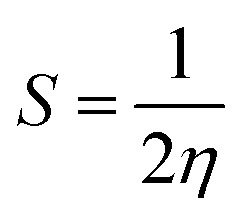

9 *ω* = *µ*^2^/2*η*

10 *χ* = −(*E*_HOMO_ + *E*_LUMO_)/2

11 IP = −(*E*_LUMO_)

12 *µ* = (*E*_HOMO_ + *E*_LUMO_)/2

13 EA = −*E*_LUMO_

**Table 3:** Quantum chemical descriptors of mitoxantrone (MTX), ethanol ammonium acetate (EAAc), ethanol ammonium butyrate (EABu), and the mitoxantrone + ethanol ammonium acetate (MTX + EAAc) and mitoxantrone + ethanol ammonium butyrate (MTX + EABu) systems

Parameters	Mitoxantrone (MTX)	EAAc	EABu	EAHx	MTX + EAAc	MTX + EABu	MTX + EAHx
LUMO (Ha)	−0.086	−0.012	−0.017	−0.018	−0.083	−0.063	−0.039
HOMO (Ha)	−0.182	−0.251	−0.161	−0.140	−0.178	−0.161	−0.140
LUMO − HOMO (Ha)	0.096	0.239	0.144	0.122	0.095	0.097	0.101
LUMO + HOMO (Ha)	−0.268	−0.263	−0.178	−0.158	−0.261	−0.224	−0.179
Ionization potential (*I*) (Ha)	0.182	0.251	0.161	0.140	0.178	0.161	0.140
Electron affinity (*A*) (Ha)	0.086	0.012	0.017	0.018	0.083	0.063	0.039
Chemical potential (*µ*) (Ha)	−0.134	−0.131	−0.089	−0.079	−0.131	−0.112	−0.0895
Electronegativity (*χ*) (Ha)	0.134	0.131	0.089	0.079	0.131	0.112	0.0895
Hardness (*η*) (Ha)	0.048	0.119	0.072	0.061	0.048	0.049	0.050
Softness (*S*) (Ha^−1^)	10.350	4.184	6.944	8.196	10.497	10.258	9.90
Electrophilicity index (*ω*) (Ha)	0.186	0.072	0.055	0.051	0.179	0.129	0.079

#### 3.4.3 Thermodynamic properties by DFT

**Fig. 10:**
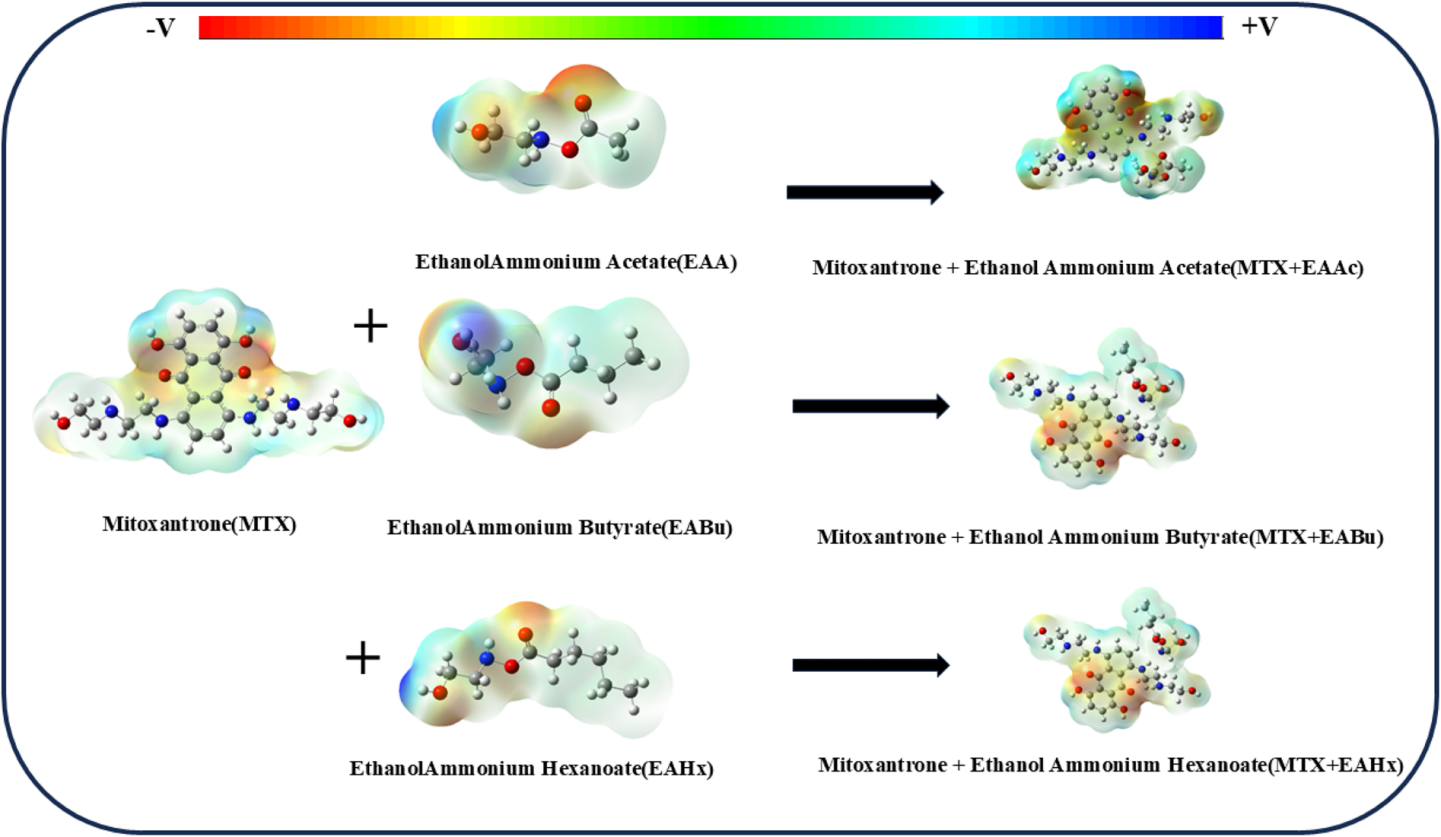
Electrostatic potential (ESP) mapping of mitoxantrone (MTX), ethanol ammonium acetate (EAAc), ethanol ammonium butyrate (EABu), ethanol ammonium hexanoate (EAHx) and their complexes mitoxantrone + ethanol ammonium acetate (MTX + EAAc), mitoxantrone + ethanol ammonium butyrate (MTX + EABu), and mitoxantrone + ethanol ammonium hexanoate (MTX + EAHx).

Thermochemical analysis provides important information about the spontaneity and stability of molecular interactions. The interactions between MTX, EAAc, EABu, and EAHx were investigated using a set of thermodynamic parameters, as shown in Table 4. The electrostatic potential maps of mitoxantrone (MTX), ethanol ammonium acetate (EAAc), ethanol ammonium butyrate (EABu), ethanol ammonium hexanoate (EAHx), and their complexes are displayed in Fig. 10.

**Table 4:** Thermodynamic parameters of mitoxantrone (MTX), ethanol ammonium acetate (EAAc), ethanol ammonium butyrate (EABu), ethanol ammonium hexanoate (EAHx), and their complexes mitoxantrone + ethanol ammonium acetate (MTX + EAAc), mitoxantrone + ethanol ammonium butyrate (MTX + EABu), and mitoxantrone + ethanol ammonium hexanoate (MTX + EAHx)

Thermodynamic properties	Mitoxantrone (MTX)	Ethanol ammonium acetate (EAAc)	Ethanol ammonium butyrate (EABu)	Ethanol ammonium hexanoate (EAHx)	Mitoxantrone + ethanol ammonium butyrate (MTX + EAAc)	Mitoxantrone + ethanol ammonium butyrate (MTX + EABu)	Mitoxantrone + ethanol ammonium butyrate (MTX + EAHx)
*ε*_o_ (Ha)	−1525.068	−438.074	−516.685	−595.291	−1963.292	−2041.962	−2120.610
*E*_zpe_ (Ha)	0.494	0.139	0.197	0.220	0.634	0.692	0.755
*ε*_tot_ (Ha)	0.526	0.149	0.209	0.240	0.679	0.739	0.771
*H*_corr_ (Ha)	0.528	0.149	0.210	0.241	0.679	0.739	0.771
*G*_corr_ (Ha)	0.427	0.101	0.156	0.184	0.545	0.601	0.640
*ε*o*+ ε*_zpe_ (Ha)	−1524.573	−437.935	−516.488	−595.071	−1962.658	−2041.270	−2119.855
*ε*o*+ ε*_tot_ (Ha)	−1524.541	−437.925	−516.476	−595.051	−1962.613	−2041.223	−2119.839
*ε*o + *H*_corr_ (Ha)	−1524.540	−437.924	−516.475	−595.050	−1962.613	−2041.223	−2119.839
*ε*o + *G*_corr_ (Ha)	−1524.640	−437.972	−516.529	−595.107	−1962.747	−2041.361	−2119.970
*E*_thermal_ (kcal mol^−1^)	330.505	93.523	131.207	153.112	425.854	463.540	490.144
Heat capacity (*C*_v_) cal mol^−1^ K^−1^)	120.011	32.733	42.101	52.099	158.645	167.367	188.211
Enthalpy change (Δ*H*) (kcal mol^−1^)	–	–	–	–	−92.870	−124.870	−155.196
Free energy change (Δ*G*) (kcal mol^−1^)		–	–	–	−84.713	−120.48	−138.991

Following complexation, mitoxantrone + ethanol ammonium acetate (MTX + EAAc) (−1963.292 Ha), mitoxantrone + ethanol ammonium butyrate (MTX + EABu) (−2041.962 Ha), and mitoxantrone + ethanol ammonium hexanoate (MTX + EAHx) (−2120.610 Ha) exhibited a notable drop in energy, showing that contact improves stability as indicated in Table 4. Interestingly, there was a greater drop in energy with mitoxantrone and ethanol ammonium hexanoate (MTX + EAHx) than mitoxantrone + ethanol ammonium butyrate (MTX + EABu), and mitoxantrone + ethanol ammonium acetate (MTX + EAAc), suggesting that mitoxantrone and ethanol ammonium hexanoate (MTX + EABu) have a stronger interaction. When compared to the electronic energies of the unbound molecules, the total energy increased with the addition of zero-point energy (*E*_zpe_). Hence, it was concluded that complexation resulted in a more thermodynamically stable system supported by the zero-point energy-corrected values (*ε*_o_ + *E*_zpe_), which showed that the complexes mitoxantrone + ethanol ammonium acetate (MTX + EAAc), mitoxantrone + ethanol ammonium butyrate (MTX + EABu), and mitoxantrone + ethanol ammonium hexanoate (MTX + EAHx) formed more stable states than their isolated components.

The sum of the electronic, vibrational, rotational, and translational contributions to the system's overall internal energy is known as the thermal energy (*E*_thermal_). Ethanol ammonium acetate (EAAc) has the lowest thermal energy (93.523 kcal mol^−1^) among the isolated compounds, while mitoxantrone (MTX) has the highest (330.505 kcal mol^−1^), as shown in Table 4. Thermal energy increased upon complex formation, rising from 425.854 kcal mol^−1^ for mitoxantrone + ethanol ammonium acetate (MTX + EAAc) to 463.540 kcal mol^−1^ for mitoxantrone + ethanol ammonium butyrate (MTX + EABu), and reaching 490.144 kcal mol^−1^ for mitoxantrone + ethanol ammonium hexanoate (MTX + EAHx). The increase in the degrees of freedom brought about by the creation of larger molecular systems resulted in the rise in thermal energy, leading to more complicated interactions and higher total energy. The production of bigger and more flexible molecular structures led to the generation of more disordered systems, as indicated by the increase in entropy values upon the interaction of mitoxantrone (MTX) with ethanol ammonium acetate (EAAc), ethanol ammonium butyrate (EABu), and ethanol ammonium hexanoate (EAHx). Compared to mitoxantrone + ethanol ammonium butyrate (MTX + EABu) and mitoxantrone + ethanol ammonium acetate (MTX + EAAc), the entropy growth for mitoxantrone + ethanol ammonium hexanoate (MTX + EAHx) was more pronounced due to its larger size and complexity, offering more vibrational modes and rotational freedom.^30,33,38^

#### 3.4.4 Electrostatic potential

Electrostatic potential must be understood in order to comprehend the molecular interactions, particularly those involving drug–ionic liquid interactions. The electric field produced by the distribution of additional charges within or between molecules provides potential to charged particles (such as an ion or a molecule with a dipole moment). This potential influences the solubility, reactivity, and binding affinity of molecules through their interactions with one another in chemical or biological systems. Computational techniques, like density functional theory (DFT), electrostatic potential maps, and molecular dynamics simulations, can be used to quantify the electrostatic potential surrounding a molecule or an ionic liquid. These illustrate how electrostatic potential is distributed around a molecule or ionic liquid (IL). To identify places that are more likely to interact with oppositely charged components of the ionic liquid, color-coded maps show regions of positive (blue), neutral (green), and negative (red) potential as shown in Fig. 10. Low ESP values generally imply a weaker electrostatic interaction between the drug and ionic liquid. This could mean less favorable binding or a reduced tendency for the drug to stay dissolved in the ionic liquid.^61–63^ The higher ESP value of the mitoxantrone + ethanol ammonium hexanoate (MTX + EAHx) complex indicates a stronger electrostatic interaction than mitoxantrone + ethanol ammonium butyrate (MTX + EABu) and mitoxantrone + ethanol ammonium acetate (MTX + EAAc) complexes, as shown in Table 5.

**Table 5:** Electrostatic potential (ESP) values of mitoxantrone (MTX), ethanol ammonium acetate (EAAc), ethanol ammonium butyrate (EABu), ethanol ammonium hexanoate (EAHx), mitoxantrone + ethanol ammonium acetate (MTX + EAAc), mitoxantrone + ethanol ammonium butyrate (MTX + EABu) and mitoxantrone + ethanol ammonium hexanoate (MTX + EAHx)

System	−V (V)	+V (V)
Mitoxantrone (MTX)	−0.108	+0.108
Ethanol ammonium acetate (EAAc)	−0.082	+0.082
Ethanol ammonium butyrate (EABu)	−0.067	+0.067
Ethanol ammonium hexanoate (EAHx)	−0.061	+0.061
Mitoxantrone + ethanol ammonium acetate (MTX + EAAc)	−0.092	+0.092
Mitoxantrone + ethanol ammonium butyrate (MTX + EABu)	−0.117	+0.117
Mitoxantrone + ethanol ammonium hexanoate (MTX + EAHx)	−0.131	+0.131

#### 3.4.5 Enthalpy(Δ*H*) and free energy(Δ*G*)

Other key indicators of the thermodynamic stability of these interactions include the changes in enthalpy (Δ*H*) and free energy (Δ*G*) upon complex formation, as listed in Table 3. The negative (Δ*H*) values for all these complexes confirm that these interactions are exothermic and thermodynamically favorable. Notably, the interaction enthalpy for mitoxantrone + ethanol ammonium hexanoate (MTX + EAHx) (–155.19 kcal mol^−1^) is significantly more negative than that for mitoxantrone + ethanol ammonium butyrate (MTX + EABu) (–124.87 kcal mol^−1^) and mitoxantrone + ethanol ammonium acetate (MTX + EAAc) (–92.87 kcal mol^−1^), highlighting a more energetically favorable interaction of mitoxantrone (MTX) with ethanol ammonium hexanoate (EAHx). Similarly, the negative (Δ*G*) values demonstrate the spontaneous nature of these interactions. The larger negative (Δ*G*) value for mitoxantrone + ethanol ammonium hexanoate (MTX + EAHx) (–138.99 kcal mol^−1^) compared to mitoxantrone + ethanol ammonium butyrate (MTX + EABu) (–120.48 kcal mol^−1^) and mitoxantrone + ethanol ammonium acetate (MTX + EAAc) (−84.713 kcal mol^−1^) further supports the conclusion that the interaction between mitoxantrone + ethanol ammonium hexanoate (MTX + EAHx) is more favorable and spontaneous.^64,65^

These findings are highly relevant to drug formulation since enhanced electrostatic stabilisation and improved solvation can mitigate premature degradation, aggregation, and precipitation, which are key factors limiting the bioavailability of salt-based anticancer drugs. Therefore, the more favored and beneficial interaction of MTX with EABu represents improved molecular stability, solubility, and controlled release potential, which are key to effective drug delivery. These results, which are supported by favorable DFT calculations and thermodynamic parameters, show that protic ionic liquids, especially EABu, can act as stabilizing co-solvents and may provide a suitable method for developing more stable, efficient, and less toxic ionic-liquid-based anticancer formulations.

## 4. Conclusions

Ionic liquids may serve as a promising platform in pharmaceutical formulations to enhance drug aqueous solubility, stability, and overall performance, particularly for salt-form drugs that exhibit low bioavailability and a tendency to aggregate in biological media. Mitoxantrone (MTX) is an example of such an anthracenedione anticancer drug, which, unfortunately, has poor aqueous solubility and a tendency to self-aggregate under physiological conditions. Herein, we have explored, at the molecular level, the interactions of MTX with three protic ionic liquids, namely, ethanol ammonium acetate (EAAc), butyrate (EABu) and hexanoate (EAHx), to investigate the effects of anionic chain length on drug solvation, stability and redox behavior. By employing a combined experimental (UV-visible absorption, fluorescence spectroscopy and cyclic voltammetry) and computational (DFT) approach, the study has demonstrated the spontaneous complexation of MTX with ILs, as well as thermodynamically favorable processes. Spectroscopic and electrochemical measurements show that fluorescence emission intensity, hypochromism, and redox reversibility increase with the length of the alkyl substituent and correlate with changes in Δ*E*. Results among the investigated systems, EAHx exhibited the most significant changes in binding and free energy (Δ*G*), indicating stronger hydrophobic, hydrogen-bonding, and electrostatic interactions with MTX. Evidence of these trends was also provided by the DFT-optimised structures and electrostatic potential maps, which showed extended charge spread and more non-covalent interactions in the MTX–EAHx complex. In general, the data presented clearly indicate a structure–property relationship for MTX–PIL systems with enhanced anionic chain length being directly related to molecular complex binding and stability. These findings, supported by molecular-level insights, provide evidence for the claimed benefits of protic ILs as functional excipients and suggest that higher molecular weight PILs may permit improved stability, solubility, and drug release performances for MTX with potential implications in the rational design of innovative ionic-liquid-based formulations as anticancer agents. The authors will consider conducting mechanistic or comparative studies in future work to demonstrate why the PIL nature or properties are necessary, as well as how the PIL characteristics offer advantages compared to the precursor materials from which they were derived, and why these properties lead to the observed experimental outcomes.

## Conflicts of interest

There are no conflicts to declare.

## Supplementary Material

RA-016-D5RA05841D-s001

## Data Availability

The authors declare that the data supporting the findings of this study are available within the article. Supplementary information (SI) is available. See DOI: https://doi.org/10.1039/d5ra05841d.
