# Supplementary material for: Decoding the interplay between protic ionic liquids and drug: spectrophotometric, electrochemical, and DFT exploration of mitoxantrone
Source: RSC Adv. 2026 Feb 19;16(11):9779–95. doi: 10.1039/d5ra05841d (PMC12917589; doi:10.1039/d5ra05841d)
Supplement: RA-016-D5RA05841D-s001 [file RA-016-D5RA05841D-s001.pdf]

Supplementary Information (SI) for RSC Advances.
This journal is © The Royal Society of Chemistry 2026

Supporting Information

Decoding Drug–Protic Ionic Liquids Interplay: Spectrophotometric, Electrochemical, and DFT Exploration of Mitoxantrone

Arti Sharma[a], Ravinder Sharma[a, b*], Ritu[a], Pamita Awasthi[a], Indra Bahadur[c*]

[a]Department of Chemistry, National Institute of Technology, Hamirpur-177005 (H.P.), India

[b]Department of Chemistry, Dr B R Ambedkar National Institute of Technology, Jalandhar–144011, India

[c]Department of Chemistry, North-West University (Mafikeng Campus), Mmabatho 2735, South Africa

*Corresponding Author email: sharmaravinder444@gmail.com, bahadur.indra@nwu.ac.za

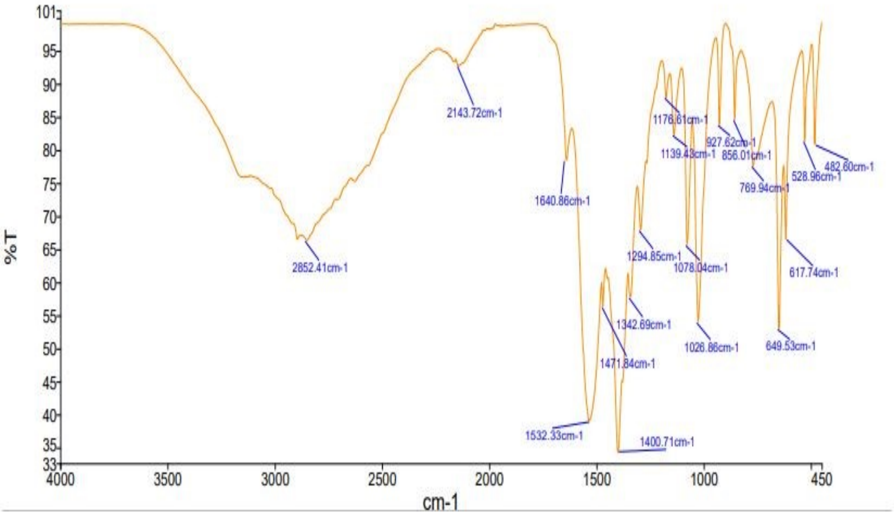

Figure S1 FTIR spectra of (a) ethanol ammonium acetate (EAAc)

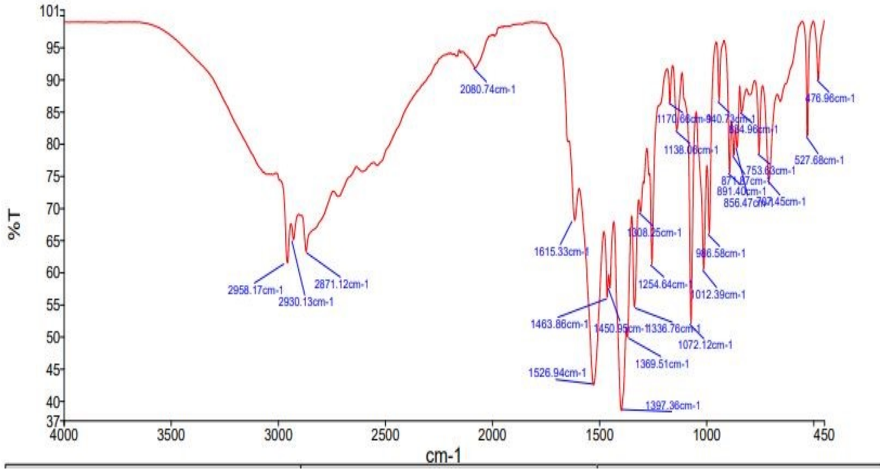

Figure S2 FTIR spectra of ethanol ammonium acetate (EABu).

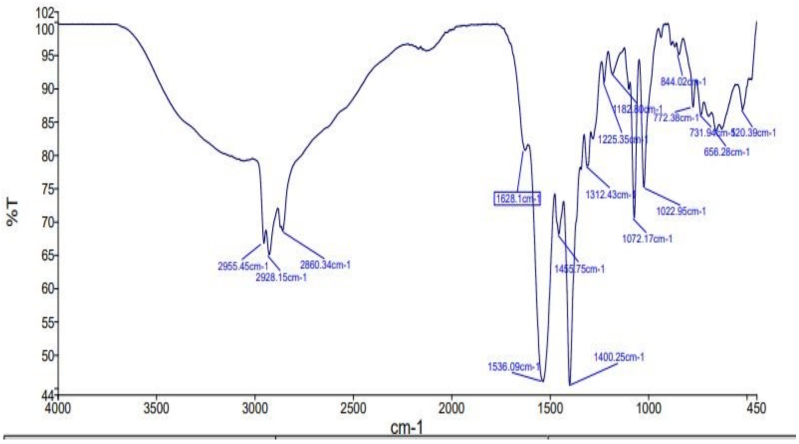

Figure S3 FTIR spectra of ethanol ammonium hexanoate (EAHx).

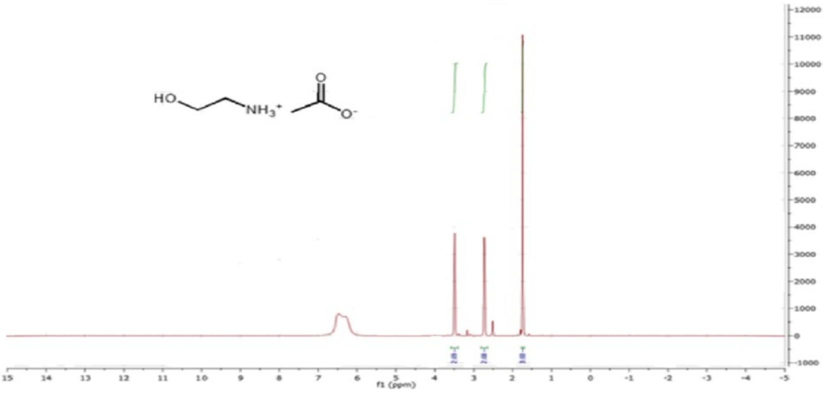

Figure S4 ^{1}H NMR spectra of ethanol ammonium acetate (EAAc). ^{1}H NMR analysis- 6.4 (Broad peak of NH_3 and OH), d = 1.9 (s, 3H); d = 2.7(t, 2H); d = 3.54 (t,2H).

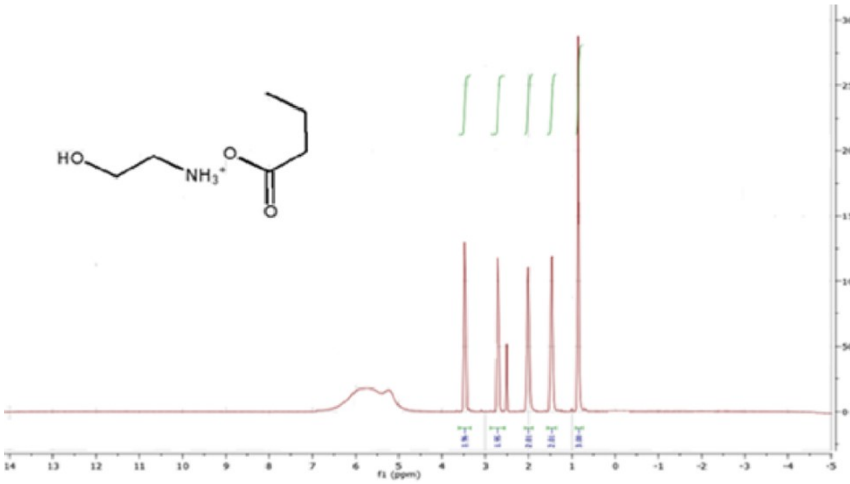

Figure S5 ^{1}H NMR spectra of ethanol ammonium butyrate (EABu).^{1}H NMR analysis- 5.5 (Broad peak of NH_3 and OH), 0.9(t, 3H); d = 1.4 (m, 2H); d = 2.0 (t,2H); d = 2.64 (t, 2H); d = 3.42 (t, 2H).[12, 29]

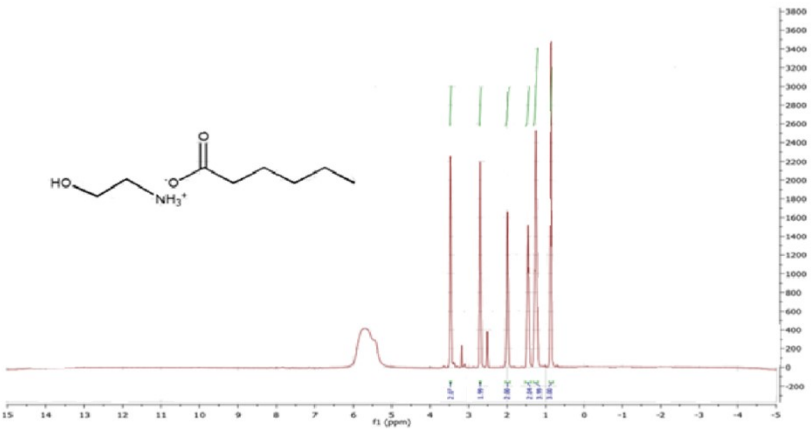

Figure S6 ^1H NMR spectra of ethanol ammonium hexanoate (EAHx). ^1H NMR analysis- 5.8(Broad peak of NH3 and OH), d = 0.9(t, 3H); d = 1.2 (m, 4H); d = 1.4 (m,2H); d = 1.99 (m, 2H); d = 2.8 (t, 2H); d = 3.5 (t, 2H).
